# Experimental and Digimat-FE Based Representative Volume Element Analysis of Dye-Mixed Colored Resin and Carbon Fiber

**DOI:** 10.3390/polym14051028

**Published:** 2022-03-04

**Authors:** Jee-Hyun Sim, Dong-Hyeon Yeo, Hyun-Sung Yoon, Seong-Hun Yu, Do-Hyun Lee, Jin-Seok Bae

**Affiliations:** 1 Department of Textile System Engineering, Kyungpook National University, Daegu 41566, Korea; maxwell02@dyetec.or.kr; 2 Korea Dyeing & Finishing Technology Institute (DYETEC Institute), Daegu 41706, Korea; yd@dyetec.or.kr (D.-H.Y.); yoon1216@dyetec.or.kr (H.-S.Y.); enviro1234@dyetec.or.kr (S.-H.Y.); lee@dyetec.or.kr (D.-H.L.)

**Keywords:** carbon fiber-reinforced plastic, resin transfer molding, interfacial adhesion, computer-aided engineering, representative volume element (RVE) model, epoxy resin

## Abstract

Recently, the automobile industry has demanded weight reduction, so research on materials is being actively conducted. Among this research, carbon fiber-reinforced composite materials are being studied a lot in the automobile industry due to their excellent mechanical properties, chemical resistance, and heat resistance. However, carbon fiber-reinforced composite materials have disadvantages, in that they are not free from color selection, and have weak interfacial bonding strength. In this study, a colored epoxy resin was prepared by mixing epoxy—which is a thermosetting resin according to the pigment concentration (0.1, 0.3, 0.5, 1.0 wt%)—and curing shrinkage. Thermal expansion characteristics were analyzed and the concentration of 0.5 wt% pigment showed the lowest shrinkage and thermal expansion characteristics. In addition, to measure the interfacial shear strength (IFSS) of the carbon fiber and the colored epoxy resin, the IFSS was obtained by performing a microdroplet debonding test, and the strength of the pigment concentration of 0.5 wt% was reduced to a relatively low level. Through these experiments, it was determined that an epoxy resin in which 0.5 wt% pigment is mixed is the optimal condition. Finally, using the composite material modeling software (Digimat 2020.0), the representative volume element (RVE) of the meso-scale was set, and interfacial properties of carbon fibers and colored epoxy resins were analyzed by interworking with general-purpose finite element analysis software (Abaqus CAE).

## 1. Introduction

Carbon fiber-reinforced plastic (CFRP) has high specific strength, specific modulus and chemical resistance, has a lower density than metal materials, and has been used in aerospace and defense industries since the 1960s due to its high mechanical properties and light weight. Since the mid-1970s its use has been expanded to various industrial fields such as sports and leisure goods, and recently, it has been widely used in various application fields such as automobiles, an industry that requires weight reduction [1,2,3,4,5]. In CFRP, the matrix is composed of thermosetting resins and thermoplastic resins, and thermosetting resins are widely used. Among these, thermosetting resins are typically epoxy, vinyl ester, phenol and the like, and the most widely used resin among them is epoxy. Epoxy resin has an irreversible property of changing its curing behavior from a viscous liquid state to a low molecular weight rubber state to a solid glass state depending on temperature [6,7,8,9,10]. Due to these characteristics it has high mechanical properties, chemical resistance, and heat resistance, and thus active research is being conducted in various applications.

In particular, it is attracting attention as a next-generation material in various mobility industries. In the case of electric battery mobility, which has recently been in the spotlight, many studies are being conducted on lightening due to the weight of the battery cell itself. CFRP parts applied to automobile parts are used in monocoque body frames, roofs, hatches, automobile seats, and dashboards [11,12,13,14].

There are three major merits that can be obtained by applying carbon composite materials to automobiles: (1) improvement in fuel efficiency, (2) improvement in the basic performance of automobiles, and (3) improvement in mileage by reducing the weight of electric vehicles (EVs) and fuel cell electric vehicles (FCEVs). As a result of analyzing the results of 10 kg weight reduction of automobiles at the Korea Institute of Science and Technology, it is predicted that fuel efficiency will improve by 2.8%, CO_2_ will decrease by 4.3%, and NOx will decrease by 8.8%. However, in the case of general CFRP, it was difficult to have a choice of color because carbon fiber was used. With the development of colored resin for resin transfer molding (RTM), it is possible to secure a consumer base through the improvement and diversification of commercial properties by applying the color diversification of existing CFRP materials to emotional materials that require design, such as vehicle interior materials [15]. However, fiber-reinforced composite materials are used in various engineering fields, the load applied to the composite material is mainly transmitted to the fibers, and the fibers break at the weakest part of the fibers. However, even in the case of fiber failure in composite materials, the load can be transmitted through the fiber/matrix interface [16,17]. Therefore, carbon fiber has a disadvantage in that it is easily broken due to weak IFSS when CFRP is manufactured by combining with a such thermosetting and thermoplastic matrix due to poor interfacial bonding strength. 

Therefore, in this study, in order to analyze the properties of composite materials used in various applications requiring light weight and color, the shrinkage rate and linear expansion coefficient of colored epoxy resin according to pigment concentration were measured using a thermochemical analyzer (TMA, TA instrument, Q400). Next, after curing the colored epoxy resin by dropping it on the carbon fiber, a microdroplet debonding test was performed to measure the IFSS of the carbon fiber and the colored epoxy resin. Finally, this simulation was performed by interlocking analysis with composite material modeling software (Digimat 2020.0) [17,18,19] and general-purpose finite element analysis software (Abaqus CAE) [11,17] to analyze the experimental results and analyze the interface characteristics between the resin and the fiber.

## 2. Materials and Methods

### 2.1. Materials

In this study, we conducted research on the production of test pieces and material analysis using low-viscosity epoxy resin (YD-128, Kukdo Chemical Industry Co. Ltd. (Seoul, Korea)) and a curing agent (4,4-Diaminodicyclohexylmethane, PACM). The pigments were inorganic pigment G1 secured in a 1:1 composition ratio of inorganic pigment Y and BL, and chlorinated Cu-Phthalocyanine organic pigment G2. Table 1 is a physical property table for resin.

After mixing an epoxy resin and a pigment to prepare a colored epoxy resin, it was dropped on carbon fiber to prepare a microdroplet debonding test specimen.

The low-viscosity resin used in this study was designed to be used appropriately at the process temperature of 130 °C inside and outside the RTM (resin injection molding) device used, the material properties were less than 400 cPs, and the tensile strength was 30 Mpa or more.

The pigment was a low-viscosity colored resin in order to ensure high-temperature stability with a weight change of less than 5% at 160 °C or higher, to minimize the dispersion stability of the colored resin through color-developing material particle size adjustment and surface treatment, and to minimize filtering by application to the RTM process. It also optimized the compounding viscosity of the pigment.

To check the color of the colored resin, the color was quantified by referring to the Fenton color and color code table, and the results are shown in Table 2 below.

### 2.2. Methods

#### 2.2.1. Manufacture of Colored Epoxy Resin

In this study, in order to analyze the interfacial properties between fibers and resins, a colored epoxy resin was produced by mixing pigments with a low viscosity epoxy resin at 0.1, 0.3, and 1.0 wt%.

#### 2.2.2. Thermal Properties Analysis

TMA (thermomechanical analyzer, TA Instrument, TMA Q400) was used to confirm the curing shrinkage and thermal expansion characteristics of the colored epoxy resin. For stable measurement, linear shrinkage was derived from Equations (1)–(3) by analyzing at a temperature of 120 °C, and a temperature increase rate of 20 °C/min using an expansion-type Quartz probe:

(1) $$\varepsilon_{L}={\left[\left(1+\nu \right)\left(\frac{\nu }{1-\nu }+1\right)\right]}^{\;-1}\left(\frac{h-h_{0}}{h_{0}}\right)$$

where $\varepsilon_{L}$ = Linear shinkage, ν = Poisson’s ratio, *h* = Specimen’s thickness.

Assuming that the resin is not compressed, ν = 0.5, and it can be expressed as Equation (2).

(2) $$\varepsilon_{L}=\frac{1}{3}\left(\frac{h-h_{0}}{h_{0}}\right)$$

Finally, the linear change was expressed as the volume change rate (εv), as in Equation (3).

(3) $$\varepsilon_{\nu }={\left[1+\frac{1}{3}\left(\frac{h-h_{0}}{h_{0}}\right)\right]}^{3}-1$$

In addition, the thermal expansion characteristics of the colored epoxy resin were calculated by calculating the coefficient of thermal expansion (CTE) for an applied load of 50 mN, a temperature increase rate of 5 °C/min, and a temperature range of 50 to 300 °C.

#### 2.2.3. IFSS of Carbon Fiber/Colored Epoxy Resin

**Figure 1:**
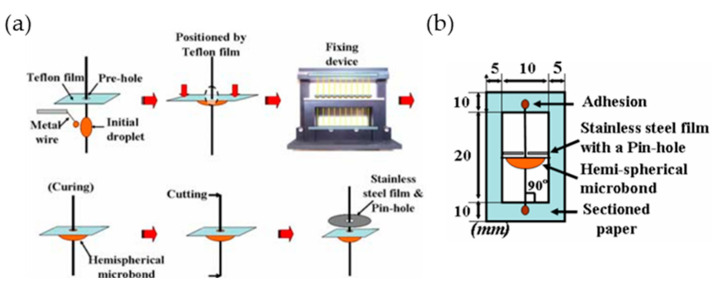
Method of (**a**) manufacturing and (**b**) measuring test piece for measuring interfacial adhesive strength between fiber and resin.

To check the interfacial bonding strength between carbon fibers and colored epoxy resins, IFSS was obtained by performing a microdroplet debonding test using colored epoxy resins according to pigment concentrations (0.1, 0.3, 0.5 and 1.0 wt%) on carbon fibers (Hyosung). Prior to this study, in order to analyze the interface characteristics between the fiber and the resin, an experiment was conducted as shown in Figure 1 to analyze the difference in the interface characteristics depending on the shape of the resin. After passing a single fiber through a Teflon film with a hole of about 30 μm, the fiber was fixed to a metal frame for fixing the fiber. An initial droplet was formed using a thin metal wire and the Teflon film was lowered slightly downward to form the droplet into a hemispherical shape.

The test piece was cured under the condition of 80 °C/30 min in an oven infused with nitrogen gas to prevent the oxidation of carbon fiber, and after cutting the fiber of the cured sample to a certain length, stainless steel with a hole of 25 μm was used. First, based on ASTM C 1239-07, place carbon fibers one by one on a polyimide tensile frame, and fix both ends with an epoxy adhesive to make a tensile specimen. A drop of nylon 6 resin was deposited on the center of the carbon fiber of the sampled tensile frame (Figure 1a). Next, pass the carbon fiber through the loading blade at 50 μm intervals, place the resin drop, and apply a load to perform the tensile test until the resin drop is broken (Figure 1b). IFSS was derived using Equation (4):

IFSS = Fmax/S (4)

where Fmax = maximum load (to debond the fiber from matrix), and S = fiber embedded area.

### 2.3. Modeling and Simulation Using CAE 

**Figure 2:**
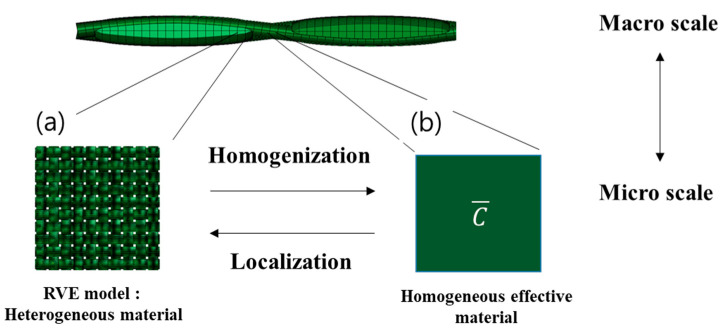
(**a**) Homogenization and (**b**) localization of RVE model.

In this simulation, a mesoscale representative volume element (RVE) was set as a model that could consider the structural properties of the woven composite material with the composite material modeling software (Digimat 2020.0) and the general-purpose finite element analysis software (Abaqus CAE). Figure 2. represents the algorithm for reconstructing the composite material with RVE. Analysis and experiments were conducted to analyze the interfacial properties of resin and fiber. Table 3 is a table that establishes basic physical property data for modeling. 

The following is the boundary condition of digimat’s RVE model:

$$u^{+}-u^{-}=\bar{\epsilon }\left(x^{+}-x^{-}\right)$$

$$\bar{\sigma }=\bar{C}:\bar{\epsilon }$$

$$\bar{\sigma }=\frac{1}{V}\int^{\;}\sigma dV$$

In the RVE model, periodic boundary conditions were applied to the RVE bound-ary such as the equation for homogenization, and the boundary of the material model satisfied the meaningless model continuity to calculate the homogenized physical properties. In this study, a representative volume element (RVE) of the meso scale was established as a model that can consider the structural characteristics of woven composites.

In the RVE model, periodic boundary conditions were applied to the RVE boundary such as the equation for homogenization, and the boundary of the material model satisfied the meaningless model continuity to calculate the homogenized physical properties.

The finite element numerical analysis model of the three models used is shown. Since all three models had an embedding length of 100 μm and a 25 μm pinhole was used for the droplet model, the contact position between the epoxy droplet and the pinhole was modeled to be 12.5 μm at the center of the fiber axis. In the hemispherical model, the pinhole contacted the upper horizontal plane of the test piece, so modeling fixed the horizontal plane in the Z-axis direction. In the inverted hemispherical test piece, the lower part of the resin was fixed by modeling because the vise tip came into contact with the stainless thin plate fixed to the lower part of the test piece. In addition, all three models were set to non-fixed fiber axis r direction and axisymmetric conditions. The load acting on the upper part of the fiber shaft was 44.49 mN, which was the average measured value of the interfacial fracture load of the hemispherical test piece with an embedded length of 100 μm prepared under the curing conditions, and this value acted on the cross-sectional area of the fiber. The physical properties of the fibers and resins were applied by applying the values used in the previous research and, based on this, the textile fibers were modeled with general-purpose finite element analysis software, and the physical properties derived from the physical properties were obtained and reflected.

**Figure 3:**
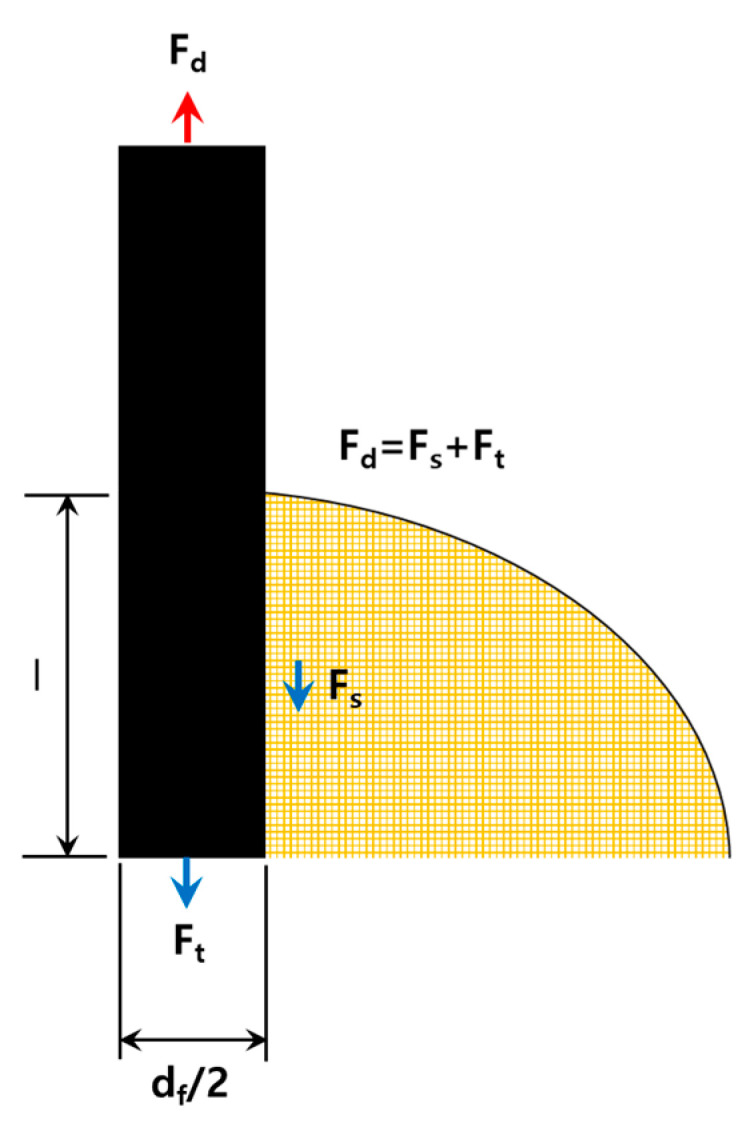
Modelling of finite element numerical analysis of adhesion.

Figure 3 is a schematic diagram of the load applied to the fiber. The load Fd applied to the upper end of the fiber is the shear load Fs acting on the interface between the fiber and the resin and the tensile load Ft acting on the lower end of the fiber acting together. Assuming the same, it can show a difference of up to 2.8 times depending on the ratio of the fiber embedding length and the fiber cross-sectional diameter.

## 3. Results

### 3.1. Thermal Properties Analysis

Table 4 shows the experimental results of volume change (shrinkage) of colored epoxy resin. The dimensional change of the colored epoxy resin specimen was considered from the point when the temperature inside the chamber reached isothermal temperature and was stabilized, and the measured dimensional change and volume change rate of the specimen were derived from Equations (1)–(3). As the pigment mixing ratio of the epoxy resin increased, the shrinkage ratio tended to decrease, but when the pigment mixing ratio was 1.0 wt%, the shrinkage ratio was higher than 0.5 wt%.

Table 5 shows the results of the linear expansion coefficient of colored epoxy resin using TMA. In the case of the epoxy resin in which the pigment was mixed in the proportion of 0.5 wt%, the thermal expansion characteristics were the lowest, and the value was reduced by about 10.5% or more compared to the case of 0.1 wt%. It was judged that the pigment had a more rigid internal structure than the case of 0.5 wt%.

### 3.2. IFSS of Carbon Fiber/Colored Epoxy Resin

**Figure 4:**
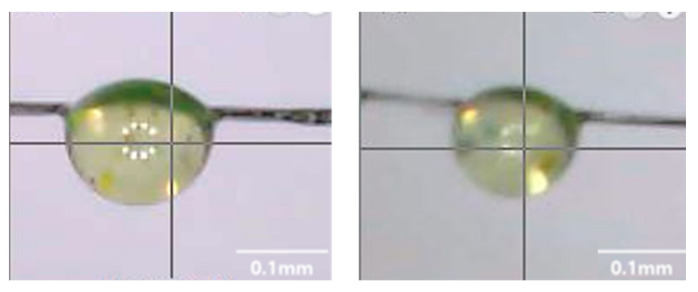
Specimen of microdroplet debonding test.

**Figure 5:**
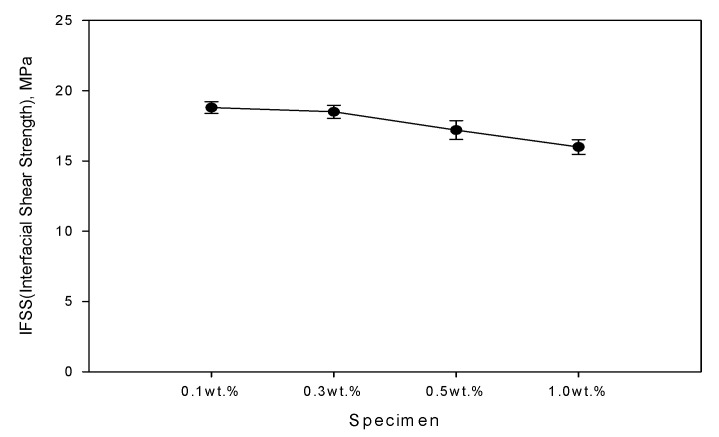
IFSS of epoxy resin according to carbon fiber and pigment concentration.

Figure 4 is a specimen prepared for a microdroplet debonding test, which was a pull-out test. Figure 5 is the result of IFSS through the microdroplet debonding test to evaluate the interfacial properties of the colored resin and carbon fiber according to the pigment mixing ratio. As the concentration of the pigment increased, the IFSS showed a tendency to decrease, and the specimen containing 1.0 wt% was measured as the lowest at 16 MPa. However, when the pigment was 0.5 wt%, the decrease in IFSS was relatively small due to the interaction and structural characteristics between the pigment and the polymer chain, and it was judged to be the most efficient formulation.

### 3.3. Results of Interfacial Properties of Simulation

**Figure 6:**
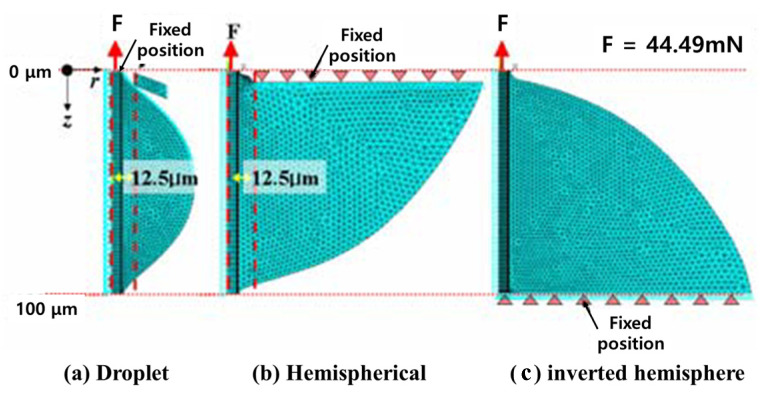
Simulation behavior according to droplet according to load.

**Figure 7:**
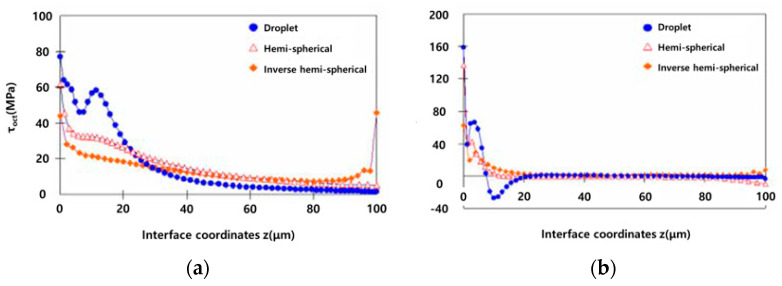
Stress graph of the simulation model along the z-axis. Graph of octahedral interfacial shear stress (**a**) τoct distribution and (**b**) angle of finite element numerical analysis models of three specimens.

Figure 6 and Figure 7 are simulation results for the interfacial bonding force of carbon fiber/colored epoxy resin. Droplet models display higher interfacial fracture loads than other models. It was also shown that the inverted hemispherical model, unlike the other two models, had a large increase in tensile stress near z = 100 μm. After that, shear-type interfacial fracture progresses were seen on the side surface of the fiber. Based on such a stress distribution phenomenon, the stress mode change and the rapid increase in stress at the interface between the fiber and the resin greatly differed depending on the model type, and the data variation in the interface shear strength evaluation of the fiber composite material was very large.

As a result of analyzing the simulation models (Figure 6), the Droplet model showed a higher concentration of τ_oct_ stress compared to the hemispherical and inverted hemispherical models at the z-axis 0 μm position, and the stress magnitude was varied from 0 to 20 μm. However, the hemispherical and inverted hemispherical specimens showed significantly similar stress distributions compared to the droplet specimens with gentle stress concentration.

The pressure was applied in the direction of the central axis of the fiber at the 10 μm area of the *x*-axis, where the tensile stress was converted into the compressive stress, thereby suppressing the interfacial fracture of the droplet specimen (Figure 7).

## 4. Discussion

This study analyzed the interfacial adhesion [20] between fibers and resins using colored low-viscosity resins and carbon fibers. First, the shrinkage rate and linear expansion coefficient of the colored epoxy resin were measured through TMA. Shrinkage and coefficient of linear expansion are important factors in the manufacture of composite materials because molding defects such as the lowering of interfacial bonding strength and the generation of cracks can appear. As the concentration of the colored epoxy pigment increased, the shrinkage rate and coefficient of linear expansion tended to be lower. In the case of shrinkage, 1.0 wt% of pigment concentration was higher than 0.3 and 0.5 wt%, and 0.5 wt% showed the lowest shrinkage. Moreover, the coefficient of linear expansion was found to be the lowest at 0.5 wt%, similar to the shrinkage rate.

The IFSS showed a tendency to decrease as the concentration of the pigment increased, and the specimen containing 1.0 wt% was measured to be the lowest at 16 MPa. However, when the pigment was 0.5 wt%, the decrease in IFSS was relatively lower than that of the pigment added at other concentrations. It was considered that the pigment acted as an impurity in the epoxy resin, and the interfacial bonding strength was lowered. Based on these experimental results, it was determined that 0.5 wt% of the pigment concentration of the epoxy resin was the optimal condition.

For the simulation of IFSS, the software (Digimat 2020.0) and the finite element analysis software (Abaqus CAE) were interlocked to model yarn and resin, and the performance was compared to derive a plan to improve interfacial adhesion.

In order to determine the interfacial adhesion, an analysis sample using a tensile strength tester was prepared, the interfacial adhesion was measured, and composite material modeling was performed based on the analyzed physical properties. Simulation was conducted to measure the durability and lifespan of the material through modeling the manufactured composite material.

As a result of the simulation, the thickness of the interface between the fiber and the resin was judged to have excellent durability and fatigue properties at the level of 0.1 mm. It was confirmed that the results similar to the actual simulation values were followed even when the durability test was performed.

Through this study, the IFSS between colored resin and carbon fiber was analyzed, and modeling and simulation were performed. When the actual experiment was conducted, it was confirmed that the simulation result value and the actual experimental value appeared very similar, and it was confirmed that the interfacial bonding of the composite material and related properties and durability could be predicted through CAE.

## Figures and Tables

**Table 1:** Resin Properties (YD-128).

EEW(g/eq)	187–205
Viscosity (cps at 25 °C)	12,000–18,000
Specific Gravity (20 °C)	1.17
Color (G)	0.5 max.

**Table 2:** Fenton color and color code table.

	L, a, b Analyze			Color Table	
	L	a	b	Fenton Color	Color Code
Result	39.47	−36.18	13.00	3435C	#006400

**Table 3:** Basic physical property DB of fibers and resins for use in Digimat modeling.

Material	Density (g/cm^3^)	Young’s Modulus (MPa)	POSSING’SRATIO	Tensile Strength (MPa)	Elogation (%)
Fiber	1.76	230,000	0.2	3530	1.5
Resin	1.1	3400	0.3		

**Table 4:** Volume change rate of colored epoxy resin through TMA.

Specimen	Specimen Thickness with Cover Glass (mm)	Initial Specimen Thickness (mm)	Shrinkage (%)	Dimensional Change, △T μm)
0.1 wt%	1.71	0.60	1.12	15.99
0.3 wt%	1.73	0.62	0.87	14.78
0.5 wt%	1.71	0.60	0.66	13.00
1.0 wt%	1.68	0.60	0.89	11.57

**Table 5:** Coefficient of linear expansion (CTE) of colored epoxy resin according to pigment concentration.

Specimen	CTE (μm/m(m °C))
0.1 wt.%	70.15
0.3 wt.%	69.25
0.5 wt.%	67.21
1.0 wt.%	67.07

## References

[1] Moon C.K., Ann C.H., Lee J.O., Cho H.H., Park J.M., Park T.W. (1993). Improvement of Mechanical Properties in Carbon Fiber Reinforced Thermoplastic Composites by Surface Modification. Polymer.

[2] Lee Y.-S., Song S.-A., Kim W.J., Kim S.-S., Jung Y.-S. (2015). Fabrication and Characterization of the Carbon Fiber Composite Sheets. Compos. Res..

[3] Kim Y.A. (2003). Carbon Fiber Composite. Phys. High Technol..

[4] Joshi S., Rawat K., Balan A.S.S. (2018). A Novel Approach to Predict the Delamination Factor for Dry and Cryogenic Drilling of CFRP. J. Mater. Proc. Technol..

[5] Xu J., Li C., Mi S., An Q., Chen M. (2018). Study of Drilling-induced Defects for CFRP Composites Using New Criteria. Compos. Struct..

[6] Lionetto F., Moscatello A., Maffezzoli A. (2017). Effect of Binder Powders Added to Carbon Fiber Reinforcements on the Chemoreology of an Epoxy Resin for Composites. Compos. Part B-Eng..

[7] Ellis B. (1993). Chemistry and Technology of Epoxy Resins.

[8] Zhandarov S., Mader E. (2005). Characterization of Fiber/matrix Interface Strength: Applicability of Different Tests, Approaches and Parameters. Compos. Sci. Technol..

[9] Sikarwar R.S., Velmurugan R., Gupta N.K. (2014). Influence of Fiber Orientation and Thickness on the Response of Glass/epoxy Composites Subjected to Impact Loading. Compos. Part B.

[10] Park S.J., Seo M.K., Lee J.R., Lee D.R. (1999). Isothermal Cure Kinetics of Epoxy/Phenol-novolac/Latent Catalyst System. J. Korean Fiber Soc..

[11] Taghia P., Bakar S.A. (2013). Mechanical behaviour of confined reinforced concrete-CFRP short column-based on finite element analysis. World Appl. Sci. J..

[12] Rajak D.K., Wagh P.H., Linul E. (2021). Manufacturing technologies of carbon/glass fiber-reinforced polymer composites and their properties: A review. Polymers.

[13] Shehab E., Meiirbekov A., Amantayeva A., Suleimen A., Tokbolat S., Sarfraz S. (2021). A Cost Modelling System for Recycling Carbon Fiber-Reinforced Composites. Polymers.

[14] Boros R., Sibikin I., Ageyeva T., Kovács J.G. (2020). Development and validation of a test mold for thermoplastic resin transfer molding of reactive PA-6. Polymers.

[15] Pimenta S., Pinho S.T. (2014). An analytical model for the translaminar fracture toughness of fibre composites with stochastic quasi-fractal fracture surfaces. J. Mechan. Phys. Solids.

[16] Na W., Lee G., Sung M., Yu W.R. (2016). Prediction of tensile and flexural strength of unidirectional CFRP considering the interfacial shear strength. AIP Conf. Proc..

[17] Tseng H.C., Hsu C.H., Chang R.Y. (2017). Long Fiber Orientation and Structural Analysis Using MOLDEX3D, Digimat and ABAQUS Simulations.

[18] Landervik M., Jergeus J. Digimat Material Model for Short Fiber Reinforced Plastics at Volvo Car Corporation. Proceedings of the 10th European LS-DYNA Conference.

[19] Trzepieciński T., Ryzińska G., Biglar M., Gromada M. (2017). Modelling of multilayer actuator layers by homogenisation technique using Digimat software. Ceram. Int..

[20] Xu B., Wei M.-Y., Wu X.-Y., Fu L.-Y., Luo F., Lei J.-G. (2021). Fabrication of Micro-Groove on the Surface of CFRP to Enhance the Connection Strength of Composite Part. Polymers.

